# Heavy metal tolerance and biosorption efficiency of *Lysinibacillus fusiformis* SKT23 novel strain for Zn and Cu removal

**DOI:** 10.1186/s12866-026-05000-z

**Published:** 2026-04-27

**Authors:** Doaa Abdullah, Tharwat E. E. Radwan, Amany M. Reyad

**Affiliations:** Botany Department, Faculty of Science, Fayoum University, Fayoum, Egypt

**Keywords:** Biosorption, *Lysinibacillus fusiformis*, Heavy metals, Wastewater treatment, Adsorption isotherms

## Abstract

Bioremediation offers a sustainable strategy for removing heavy metals from water by transforming toxic pollutants into less hazardous forms. In this study, a highly metal-tolerant bacterium, *Lysinibacillus fusiformis* strain SKT23 (accession PX488931), was isolated from contaminated soil in Fayoum, Egypt, and identified via morphology, biochemical tests, and 16S rRNA sequencing. The strain exhibited optimal growth at 50 ppm Zn (pH 7, 30 °C) and 50 ppm Cu (pH 8, 30 °C) in mineral salt medium, with growth monitored through optical density and protein content. SEM, TEM, and EDX analyses revealed cell surface modifications, intracellular and extracellular metal deposition, and precipitate formation under metal exposure. The adsorption performance of the prepared material was assessed through isotherm modeling and treatment of real wastewater. Isotherm modeling showed that Cu^2^⁺ and Zn^2^⁺ biosorption onto bacterial biomass followed Freundlich and Langmuir models, with n values (2.22–2.38) indicating favorable adsorption and qm values up to 120 mg/g, confirming the strong affinity of metal ions toward bacterial cell surface binding sites. In synthetic solutions, removal reached 98 ± 0.5% for Cu and 92 ± 0.7% for Zn at 50 mg/L, while in real wastewater it remained 88 ± 1.0% and 85 ± 1.2%, respectively. These findings demonstrate that *L. fusiformis* SKT23 possesses high metal tolerance, robust adsorption capacity, and effective removal efficiency in both controlled and complex environments, highlighting its practical potential for sustainable bioremediation of Cu and Zn contaminated water.

## Introduction

Environmental pollution is a worldwide problem; growing soil contamination over the past 20 years has harmed both the environment and living things [1]. Both natural processes and human activity contribute to soil contamination [2]. The later comprises organic compounds, metals (both trace and heavy), and radioactive waste, while the former includes floods, tsunamis, and volcanic eruptions [3, 4]. Heavy metals can cause toxicity by altering a number of biological reactions at the cellular and molecular level, including the inhibition of enzymes, blocking active sites or functional structures of metabolically important molecules, displacing or substituting essential elements, and disrupting membrane integrity [5–7]. Because they are not degraded or removed, these hazardous metals have a long-lasting effect on the environment [8]. They can spread and accumulate throughout the food chain after being released into the environment [9]. Additionally, they can go in the human body by airborne transmission or direct skin contact, which poses serious dangers to human health [10, 11]. Certain metals, like arsenic, copper, nickel, iron, etc., are beneficial at low concentrations [8, 12], Several of these HMs have vital functions in living organisms' biochemical, physiological, and metabolic activities. They also function as micronutrients, enzyme cofactors, and regulate the cell's pressure gradient [13]. At high concentrations, they become cytotoxic and carcinogenic for the cells, especially after prolonged exposure [14–16]. Heavy metals operate by interfering with the organs' ability to function. The most common symptom of infected bodies is organ dysfunction,zinc, for instance, can lead to serious damage to the heart, brain, kidney, gastrointestinal tract, and respiratory system [17].

Zn metal is a transition element that is frequently found on the planet's surface and is essential to almost all life things [18–20]. This shiny, brittle, blue-white in colour metal is solid at the standard temperature. It is commonly thought of as a slightly reactive metal in terms of its reactivity with oxygen and other metals, and it readily becomes moldable and flexible when heated to temperatures above 110 °C [21]. Zinc metal has a vital role in the control of many biological functions and metabolic processes in all human tissues [22–24]. However, too much zinc can have negative health effects, such as cramping, skin rashes and itching, vomiting, and gastrointestinal distress [25].

Copper concentrations in natural water usually range from 4 to 10 µgL⁻^1^; most of this is associated with organic molecules, whereas soils typically contain 50 ppm of Cu [26]. Cu substances are needed in many agricultural and manufacturing operations, and they may discharge to ecosystems and end up in various water systems [27]. Copper is an integral portion or cofactor for several proteins, commonly known as oxidoreductases, that participate in fundamental biological activities, such as superoxide dismutases (SODs), cytochrome C oxidase, L-ascorbate oxidase, ceruloplasmin, and other enzymes [28]. Although copper is necessary for the metabolic processes of mammals, excessive consumption of the element can have serious adverse effects, including elevated blood pressure, fast breathing, damage to the kidneys and liver, seizures, cramping, nausea, and even death [25, 29]. The accumulation of Cu (II) ions in the brain, skin, and pancreas, among other bodily organs, can lead to serious toxicological problems and even damage, particularly to the liver, kidneys, and respiratory system [26, 29, 30]. When it comes to removing heavy metals, the biomass of microbes is frequently mentioned as having greater biosorption ability than other biosorbents [31–33]. Among the various techniques for taking out these metals, biosorption employing different biomaterials is a popular approach that is both ecologically acceptable and adjustable to metal concentrations and conditions [34]. Bacterial biomass has been thoroughly researched for biosorption and is one of the most easily and affordably produced microorganisms [35, 36]. Previous investigations have examined a number of bacterial species, including *Lysinibacillus varians* [37–39], *Microbacterium* sp. D2-2, *Bacillus* sp. C9-3, *Staphylococcus xylosus*, *Paraclostridium bifermentans* G3, *Pseudomonas*, and *Ochrobactrum* [40, 41]. Bioadsorption, bioreduction, and bioaccumulation are among the microbial detoxification techniques that have been shown to be efficient in reducing the toxicity of Cd, zn, and Cr [42, 43]. Unfavorable conditions can result in stress reactions that exhibit recognizable changes in the structure and organization of bacterial cells. Exposure to dangerous metals, metalloids, and organics, as well as highly acidic or alkaline pH levels and unfavorable temperatures, are examples of these circumstances [44, 45]. According to Demirbas [46], the main factors influencing the bio-bloc's efficacy in the adsorption process are temperature, contact time, adsorbent dose, pH, and metal concentration. Surface precipitation, ion swap, complexation, and adsorption are the main processes in the biosorption process [47]. The multi-step process of biosorption consists of adsorption, ion exchange, complexation/chelation, and external precipitation [48]. In recent years, microbial bioremediation has gained considerable attention as an eco-friendly and cost-effective approach for the removal of heavy metals from contaminated water. Bacterial biomass contains a wide range of functional groups capable of binding metal ions through ion exchange, complexation, and electrostatic interactions, making biosorption one of the most promising techniques for wastewater treatment [49]. Among different microorganisms, Gram-positive bacteria, particularly members of the genera *Bacillus* and *Lysinibacillus*, have shown high resistance to toxic metals and strong adsorption capacity due to the presence of teichoic acids, peptidoglycan, and surface functional groups in their cell walls. Recent studies have reported efficient removal of Cu (II), Zn (II), Pb (II), and Cd (II) using bacterial biosorbents, with adsorption performance strongly influenced by environmental conditions, metal concentration, and surface chemistry of the biomass. Despite these advances, the search for new metal-tolerant strains with higher adsorption capacity and better performance in complex wastewater systems is still required. Although many studies have evaluated biosorption in synthetic solutions, fewer investigations have examined the efficiency of newly isolated bacterial strains under both controlled and real wastewater conditions, combined with detailed structural and isotherm analyses. In addition, the adsorption behavior of Cu and Zn using *Lysinibacillus fusiformis* has not been extensively studied, particularly with respect to metal tolerance, microscopic characterization, and equilibrium modeling. Therefore, the present study aimed to isolate and characterize a highly metal-tolerant strain of *Lysinibacillus fusiformis* from contaminated soil and to evaluate its potential for the removal of Cu and Zn from synthetic solutions and real wastewater using adsorption isotherms and microscopic analysis, providing new insights into its applicability for sustainable bioremediation.

## Materials and methods

### Chemicals and preparation methods

Analytical-grade chemicals and tools were utilized in the preparation of the various metal solution concentrations, which included the following: zinc reference solution (1000 mg/L) and copper reference solution (1000 mg/L) from Merck, Germany. To cultivate and separate bacteria, nutrient agar and mineral salt medium are also utilized. Biosorption tests were conducted in a metal aqueous solution that had been double distilled.

### Bacterial isolation

A sample was collected from a heavy metal-polluted area in Fayoum, Egypt. 100 microliters of the resulting solution were spread out on agar medium with varying metal concentrations and incubated for two days at 30 °C after one gram of each dry contaminated dust was dissolved in 50 millilitres of sterilized water. Morphologically different colonies were selected by repeatedly growing them on nutrient agar dishes after the isolate most resistant to the greatest amount of metal was chosen To be used in upcoming studies, the pure target isolate was kept at −80 degrees Celsius.

### Characterization and bacterial isolate identification

The strain was identified for startup identification using Gram staining. The isolated strain was also identified by biochemical characterization, which included tests for urease, hydrosulfide production, indole, and citrate, as well as tests for catalase [50], nitrate reduction [51], β-galactosidase [52], methyl red, gelatinase [53], oxidase [54], amylase, urease, and sugar fermentation.

Standard bacterial techniques were employed to extract the genomic DNA of the isolated bacterium for molecular identification. Briefly, pure bacterial colonies were inoculated into Luria-Bertani (LB) broth and incubated at 37 °C for 18–24 hours to achieve high cell density. The cells were harvested by centrifugation and washed multiple times with sterile phosphate-buffered saline (PBS). Cell lysis was performed using a combination of lysozyme treatment and SDS to disrupt the cell wall and membrane, followed by protein removal using Proteinase K and phenol:chloroform:isoamyl alcohol extraction. The genomic DNA was precipitated with cold ethanol, washed with 70% ethanol, air-dried, and finally dissolved in Tris-EDTA buffer for downstream applications [55].

For 16S rRNA gene amplification, PCR reactions were performed using R1 (GGT TAC CTT GTT ACG ACT T) as the reverse primer and AGA GTT TGA TCC TGG CTC AG as the forward primer. Each 25 µL PCR mixture contained 10–50 ng of template DNA, 0.2 µM of each primer, 200 µM dNTPs, 1× PCR buffer with MgCl₂, and 1 U Taq DNA polymerase. The amplification was carried out in a thermocycler under the following conditions: initial denaturation at 95 °C for 5 min; 35 cycles of denaturation at 95 °C for 30 s, annealing at 58 °C for 30 s, and extension at 72 °C for 90 s; followed by a final extension at 72 °C for 7 min. PCR products were analyzed on 1.5% agarose gel stained with GelRed and visualized under a UV transilluminator to confirm the expected ~1500 bp band. The purified PCR products were submitted to SIGMA-Biotech (Constance, Germany) for Sanger sequencing. The resulting sequences were analyzed and aligned against the NCBI GenBank database (www.ncbi.nlm.nih.gov) using BLASTn to identify the closest bacterial homologs and confirm the identity of the most resistant isolate.

### Optimization of the growth conditions

Bioremoval of heavy metal (H.M.) by* Lysinbacillus* in aqueous medium was detected. The bioremoval studies were carried out in 250 mL Erlenmeyer flasks with 100 mL of sterilized MSM added with different concentrations of H.M. Then, it was inoculated with 200 µL of each bacterial inoculum for 24 hrs. Using spectrophotometry, the growth of cells in liquid media was assessed by monitoring the culture optical density at 600 nm for 24 hrs*.* Uninoculated media with the same amount of H.M. were used as control.Experiments at different temperature degrees (20, 30 and 40 ºC), different pH values (3, 4, 5, 6, 7, 8, and 9), the contact time (2, 4, 6, 8, 12, 24, and 48 hrs), and different concentrations; 50, 100, 150, 200, and 250 ppm of H.M were examined for their impacts on bacterial growth. Growth determination by monitoring the culture optical density performed in line with protein content of the bacterial colonies in the same concentration*.* All the experiments were done in triplicates.

### Quantitation of proteins with Bradford assay

The Bradford assay [56] was used to estimate the protein content. Coomassie blue stain forms compounds with cationic and nonpolar hydrophobic protein side chains in acetic solutions. Consequently, the maximum absorption moves from 430 nm to 595 nm.

### Determination of metal removal efficiency

The metal solutions after the biosorption process were collected in order to assess the removal rate of metal ions by biosorbent. Biosorption capacity (qe) [57], the amount of metal adsorbed per gram of biosorbent, can be calculated at equilibrium in mg/g as follows:

$$\mathrm{qe} = \frac{\left({C}_{o}-{C}_{e}\right)}{m}\times V$$

where C0 is the initial concentration of metal ions in the solution (mg/L), Ce is the equilibrium concentration of metal ions in the solution (mg/L), V is the volume of solution (in litres), and m is the mass of biosorbent applied, in grams. Metal uptake can also be expressed as percentage of metal removal, given as:

$$\mathrm{Removal} \left(\%\right)=\frac{\left({C}_{o}-{C}_{e}\right)}{{C}_{0}}\times 100$$

The Bioremoval Efficiency tests were performed in Erlenmeyer flasks with a capacity of 250 mL. Initially, a pre-culture of *L. fusiformis* in nutrient agar medium was added to 100 mL of autoclaved MSM. Zn-supplemented medium had a pH 7, while Cu-supplemented media had a pH 8. The samples were shaken for 24 hours at 30 ºC and 150 xg for each metal. The concentrations of 50 mg/L of each metal ion were used to study bioremoval separately. The mixture underwent a 20-minute centrifugation at 4,000 xg following the reaction. Cu and Zn contents in the solution were ascertained using ICP (Perkin Elmer optical spectrometer [OES], Optima 5300 DV).

### SEM–EDX analyses

Bacterial biomass was subjected to SEM examinations both before to and following loading with metal solutions. SEM gives topographical and elemental data of the solids with a nearly infinite depth of field, enabling different specimen portions to remain in focus at the same time for characterizing bacterial biomass. Additionally, higher magnification for closely spaced materials is made possible by high resolution in SEM. Not only can it produce a real, clear image, but it can also be used to determine the topographical features of bacterial biomass both before and after metal loading [58, 59]. To examine the metal adhesion on the bacterial cell surface, EDX elemental analysis was offered.

### TEM examination

The metal in the biomass was detected using transmission electron microscopy (TEM). Sodium phosphate buffer saline (0.1 M, pH 6.8) was used to fix bacterial cell samples in mixture of glutaraldehyde (1%) and paraformaldehyde (2%). Following a 24-hour fixation interval at 4 ºC, the cells were washed in a new buffer, and the samples were centrifuged for five minutes at 4000 xg. The specimens were dehydrated in graded alcohol solutions and embedded in CY 212 araldite following multiple washing in sodium phosphate buffer. Using a transmission electron microscope (Model JEM 2100 F) to examine the grids. 60–80 kV was the operating voltage [60].

### Adsorption isotherm study

Adsorption equilibrium data were analyzed using Freundlich and Langmuir isotherm models to evaluate the biosorption of Cu^2^⁺ and Zn^2^⁺ onto bacterial biomass. The equilibrium adsorption capacity (qₑ) and equilibrium concentration (Cₑ) were calculated from batch experiments. Freundlich constants (Kf and n) were obtained from the linear plot of log qₑ versus log Cₑ, while Langmuir constants (qₘ and KL) were calculated from the linear plot of Cₑ/qₑ versus Cₑ [61, 62].

### Comparative study of Cu(II) and Zn(II) removal from synthetic and real wastewater using bacterial biomass in Fayoum, Egypt

Batch biosorption experiments were conducted to evaluate the removal of Cu (II) and Zn (II) from both synthetic aqueous solutions and real wastewater using dried bacterial biomass of *Lysinibacillus fusiformis* SKT23. The biomass was harvested from actively growing cultures, washed three times with sterile deionized water, oven-dried at 60 °C until constant weight, and ground to a uniform particle size (~100 µm). Synthetic solutions were prepared by dissolving analytical-grade CuSO₄·5H₂O and ZnSO₄·7H₂O in deionized water and spiked to a final concentration of 50 mg/L for each metal. Real wastewater samples were collected from the intake of the Qahafa Wastewater Treatment Plant in Fayoum, Egypt, using 2-liter polyethylene bottles. Samples were centrifuged and filtered through 0.45 μm Millipore membrane filters before being spiked till 50 mg/L of each metal and adjusted to optimal pH and temperature conditions. For the biosorption experiments, 50 mL of each metal-containing sample was contacted with 0.04 g of bacterial biomass in 100 mL Erlenmeyer flasks and equilibrated for the optimal contact time on a mechanical shaker. After incubation, the biomass was separated by centrifugation, and the supernatant was filtered prior to determination of residual metal concentrations using inductively coupled plasma optical emission spectroscopy (ICP-OES). All experiments were performed in triplicate to ensure reproducibility.

### Statistical analysis

In triplicate metal biosorption tests, the mean ± standard deviation (SD) was calculated. R program was used to do statistical analysis [63]. Statistical significance is defined as a *P* value < 0.05.

## Results and discussion

### Bacterial characterization and identification

**Fig. 1:**
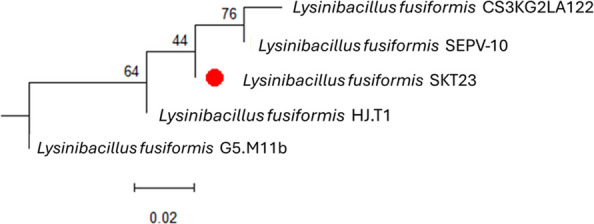
Phylogenetic tree of *Lysinibacillus fusiformis* sequences constructed using MEGA 12. The red-dotted branch indicates the sequence obtained in this study (*Lysinibacillus fusiformis* SKT23), compared against other *L. fusiformis* sequences retrieved from GenBank. The tree shows the relative genetic similarity among the sequences. Bootstrap values (percentages) are indicated at branch nodes. Scale bar represents 0.02 nucleotide substitutions per site

The bacterial strain that was shown to be the most metal-resistant could withstand up to 250 mg/L. To fully comprehend the morphological and biochemical properties of the bacterial isolate, a variety of morphological and biochemical tests were conducted, as indicated in Table 1. The bacterial cells are non-motile, Gram-positive bacilli that produce spores. It showed negative results for the synthesis of H_2_S, amylase, β-galactosidase, methyl red and oxidase, nitrate synthesis and gelatinase. Meanwhile, positive results were obtained from the tests for urease, gelatinase, catalase, citrate utilization, and indole generation (Table 1).The ability of the bacterial isolate to use glucose, cellobiose, sucrose, and casein as carbon sources was also shown at the same time. The National Center for Biotechnology Knowledge's Blastx tool (BLAST) was used to match the DNA sequences to unknown sequences. This bacterium is closely linked to the species *fusiformis* and was a member of the genus *Lysinbacillus*, as is evident. In Fig. (1), the phylogenetic tree displayed and the sequence was submitted to GenBank under accession number PX488931.

**Table 1:** Bacterial characterization

Reaction	Result	Reaction	Result
Morphological characters		Fermentation of sugars	
Gram staining	Positive	D-Glucose	Positive
Motility	Non- motile	Sucrose	Positive
Cell shape	Rod-shaped	Fructose	Positive
Endospore formation	Positive	Lactose	Positive
Spore position	Bulging,round,terminal	Maltose	Positive
Colony character	Irregular	Cellobiose	Positive
Biochemical characters		Lactose	Positive
Enzyme profile		Casein	Positive
β-galactosidase	Negative		
Nitrate reduction	Negative	Other tests	
Urease	Positive	Citrate utilization	Positive
Gelatinase	Positive	H_2_S production	Negative
Catalase	Positive	Indole production	Positive
Amylase	Negative	Methyl red	Negative
Oxidase	Negative	Voges-proskauer	Positive

### Growth optimization and H.M removed by *Lysinbacillus*

According to Demirbas [46], the main factors influencing the bio-bloc's efficacy in the adsorption process are temperature, contact time, adsorbent dose, pH, and metal concentration. Surface precipitation, ion swap, complexation, and adsorption are the main processes in the biosorption process. The impact of Zn and Cu concentrations, temperatures and pH values was investigated in order to determine the optimum growth conditions of *L. fusiformis*.

### Effect of solution pH

**Fig. 2:**
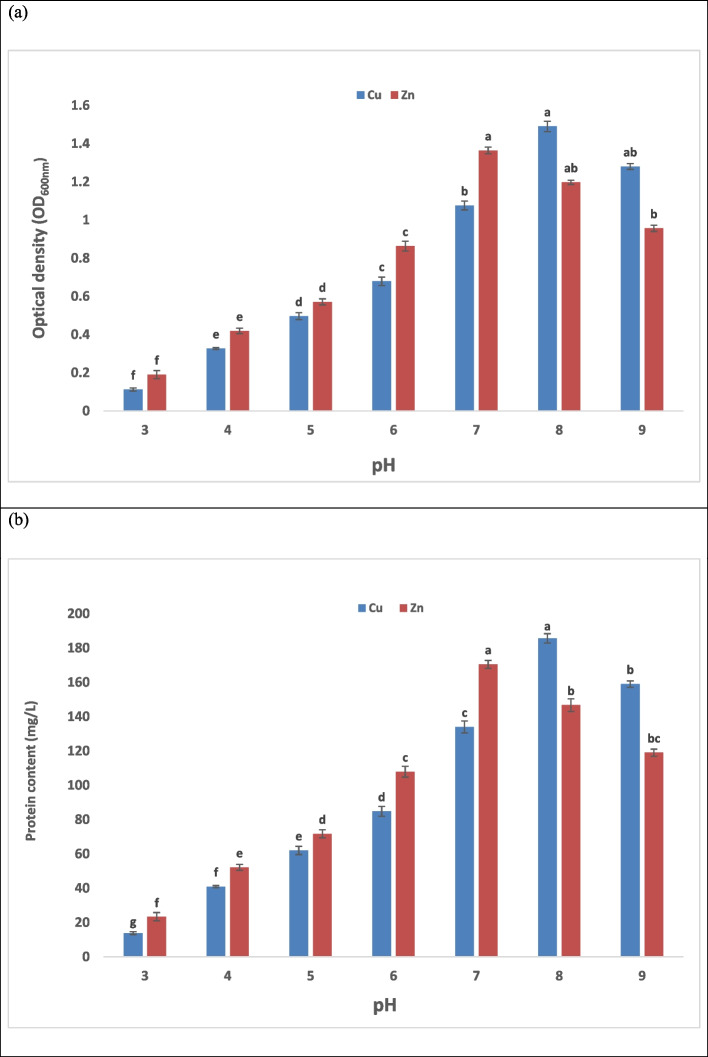
Effect of different pH on bacterial growth, where (**a**) represents optical density (OD_600_) and (**b**) represents protein content(mg/L). The data represent the means of three replicates, and the error bars display the means' standard errors

The surface charge of the bacterial cell wall, the solubility of metals, the ionization of functional groups, and the speciation of heavy metals in solution are all significantly impacted by pH [64]. When the pH changes, the basic and acidic groups on the cell wall undergo protonation and deprotonation [65]. The pH has a major influence on the chemistry of the metal's solution, the activity of functional groups on the cell wall (such amino groups and carboxylate), and the challenge of metallic ions for the binding site [66]. The investigation's pH values were altered between 3 and 8,the results for the optical density (OD_600nm_) and protein content (mg/L) were determined (Fig. 2). Our study's findings demonstrated that all metal ions have a very low biosorption capacity at low pH (< 5.0) because high concentration of hydrogen ions (H^+^) fights with metal ions at sorption sites, which repels the metal ions from the bacterial cell and renders adsorption unfavorable [67]. In order to reduce the positive charge density on the adsorbent and promote a greater metal absorption, OH⁻ improves the deprotonation reaction. The availability of additional negatively charged cells increases with pH [68]. As the pH increased, it was found that the optical density and protein content for bacteria incubated with Cu and Zn increased dramatically, as Fig. 2 (a & b) illustrates. At pH 3 and 4, both the protein content and optical density were clearly inhibited, while at pH 5, low cell growth was demonstrated in the case of treatment with Cu and Zn. At the same time, moderate bacterial growth and protein content were demonstrated at pH 6 and 8 for treatment with Zn, whereas pH 6 and 7 for treatment with Cu. It was found that the maximum optical density and protein content values for Zn (II) at pH 7 were 1.364 and 170.5 mg/L, respectively, after 24 hrs of incubation, whereas the maximum optical density and protein content values for Cu (II) at pH 8 were 1.49 and 185.6 mg/L after 24 hrs of incubation. Results indicate that pH=8 is the optimum for Cu (II) uptake, which agrees with that of Ozdemir et al. [69], El-Shanshoury et al. [70]; Bilyaminu et al. [71]. While pH 7 was optimum for Zn (II) uptake, which also agrees with the study of Bilyaminu et al. [71].

### Effect of temperature

**Fig. 3:**
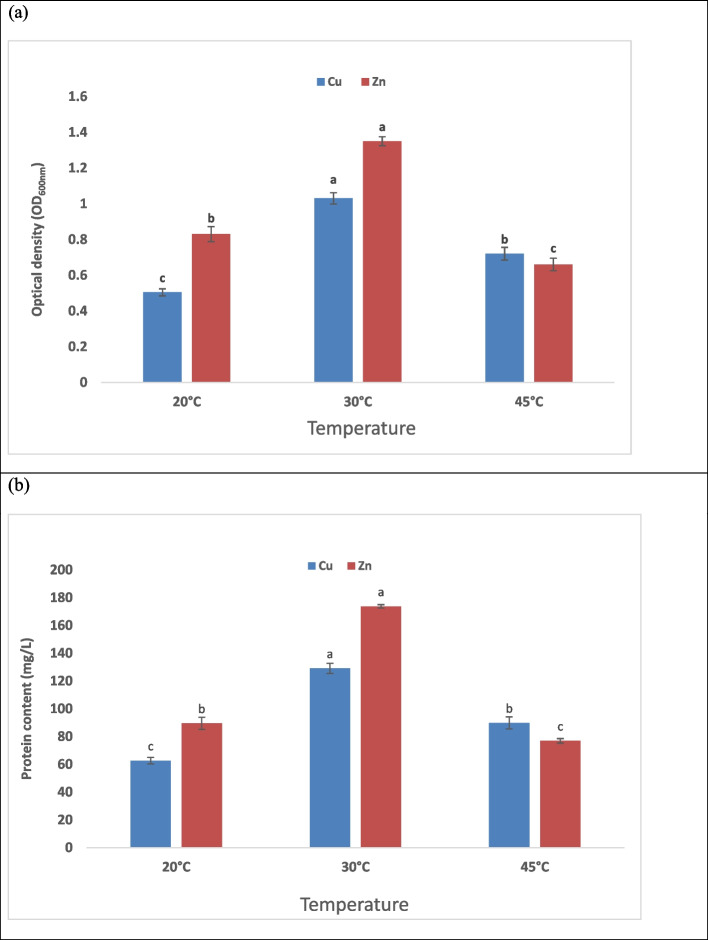
Effect of different temperature values on bacterial growth where (**a**) represents optical density (OD_600_) and (**b**) represents protein content (mg/L). The data represent the means of three replicates, and the error bars display the means' standard errors

Temperature is a major factor in metal biosorption because it changes the rate, extent and the interaction between adsorbate and adsorbent [57]. According to Arivalagan et al. [65], the fact that uptake increases as temperature rise indicates that process is an endothermic process. Heavy metals biosorption is typically altered by temperature because of the rise in the solute `s surface activity and kinetic energy, but higher temperatures can also destroy some of the binding sites that are accessible to metal ions [72]. Moreover, the obtained results demonstrated effect of different temperature degrees on the growth. The maximum optical density (1.35 & 1.03) and protein content (173.6& 129.1 mg/L) for Zn and Cu respectively were demonstrated at 30 ºC after 24 hrs of incubation. Data in Fig. (3) show the effect of different temperatures on the growth of *Lysinbacillus* treated with Zn and Cu respectively. At temperatures (20 & 45 °C) clear inhibition in the optical density (0.83, 0.504, 0.66, and 0.72) and protein contents (89.5, 62.6, 76.9, and 89.8 mg/L).

### Effect of contact time

**Fig. 4:**
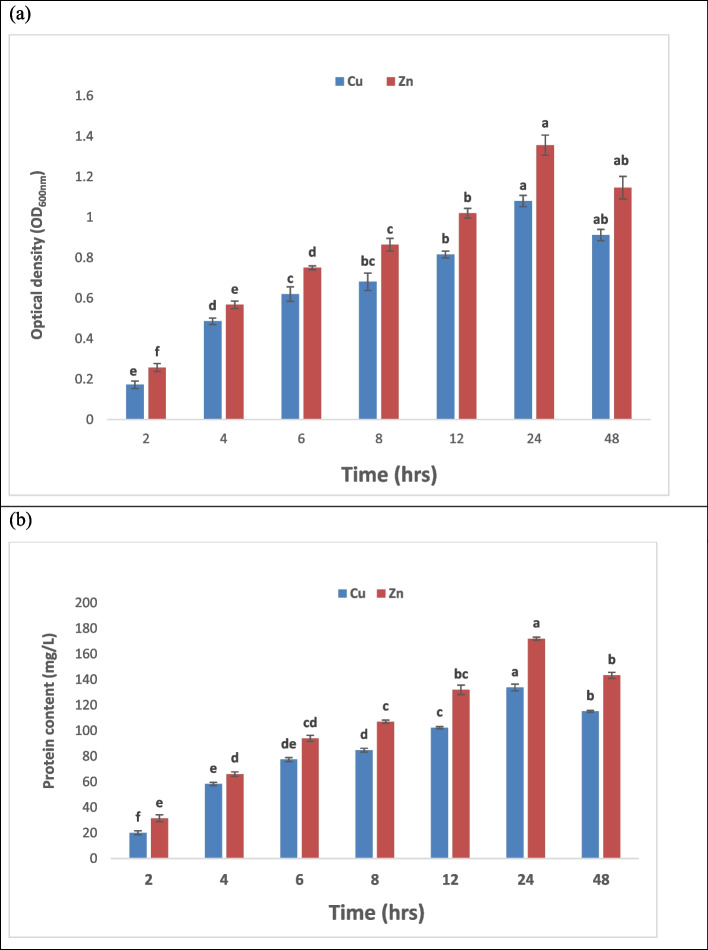
Effect of different time values on bacterial growth where (**a**) represents optical density (OD_600_) and (**b**) represents protein content (mg/L).The data represent the means of three replicates, and the error bars display the means' standard errors

The contact time between the bacterial cells and the metal solutions has a significant impact on the metal uptake. As time passed, metal ions progressively filled empty spaces, and the bacterial cell walls, lacking free active sites or having very few of them, led to the sites eventually reaching equilibrium [73]. The sorption equilibrium time shows the sorption-desorption processes that take place on the biomass surface following the saturation of metal ions [74]. In the biosorption process, contact time is one of the fundamental components. Between 2 and 24 hrs. The impact of contact time on bacterial growth with the presence of Cu (II) and Zn (II) ions from solution was investigated at different intervals. Contact duration is crucial for bacterial growth and protein content, as shown in Fig. (4). Up to 24 hours of increased contact, time results in an increase in Zn and Cu uptake. The results are displayed in Data, where it is evident that microbial biomass required 24 hours of equilibration to achieve maximum uptake of Zn (II) and Cu (II). Data in Fig. (4) show that the maximum uptake for Cu (II) and Zn (II) occurred after 24 hrs, this is in agreement with the study of El-Shanshoury et al. [70] who mentioned that, maximum bio-sorption rates for Zn(II) and Cu(II) by *Enterobacter* sp. could be obtained at 24 hrs. Also Arivalagan et al. [65] show similar results with *Bacillus cereus* for uptake of Cu (II) and results obtained by Wani et al. [75] agree with our study results.

### Effect of initial concentration of metal ions

**Fig. 5:**
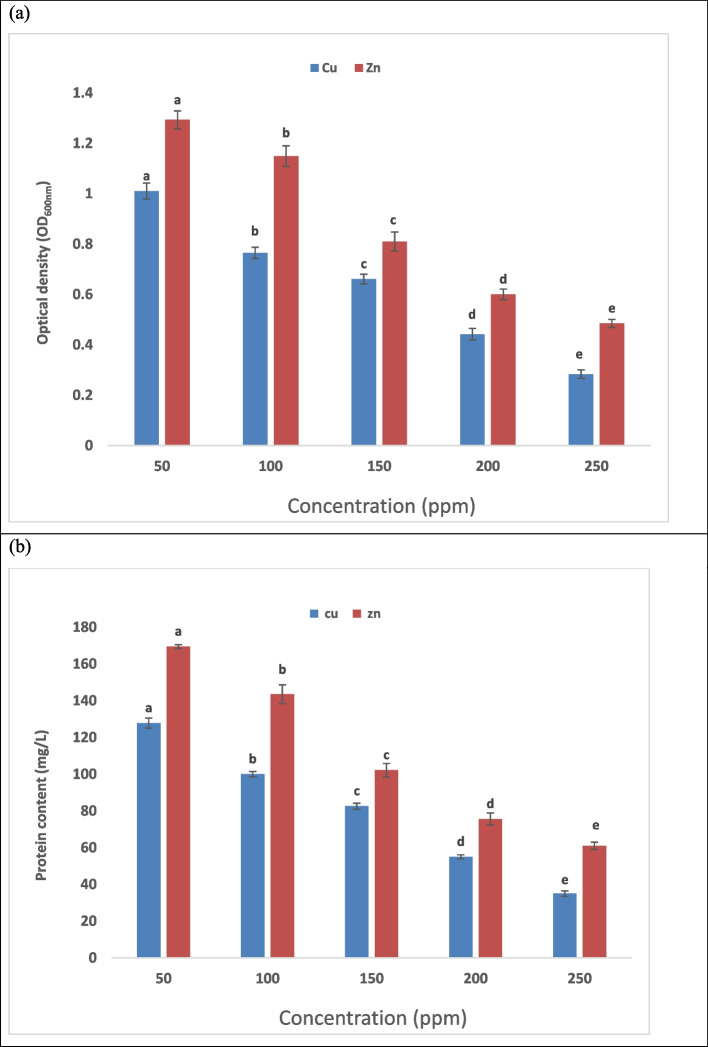
Effect of different Zn and Cu Concentrations on bacterial growth where (**a**) represents optical density (OD_600_) and (**b**) represents protein content(mg/L). The standard errors of the means are shown by the error bars, and the data are the means of three replicates

An essential factor in the biosorption process is the initial concentrations of metal ions [76]. A rise in metal ion concentrations results in an increase the number of metal ions per unit mass of biomasss because the initial concentration of metal ions reduces the sorption percentage while providing the driving force to needed to get over the resistance to the transport of metal ions between liquid and hard phases. An increase in metal ion concentrations leads to an increase in the number of metal ions per unit mass of biomasses [77]. Every metal ion in the solution may improve the interaction between metal ions and bacterial binding sites at lower metal ion concentrations, increasing the efficiency of biosorption. Because of the saturation of possible binding sites, absorption capacity is nearly constant at increasing concentrations [78]. The data in Fig. (5) demonstrate how *Lysinbacillus fusiformis* growth was impacted by zinc concentrations (50 - 250 ppm). The highest protein content (169.5 mg/L) was found after 24 hrs at same dose of zinc, while the maximum optical density (1.293) was recorded at 50 ppm of zinc after 24 hrs of incubation. The optical density and protein content clearly decreased at concentrations higher than this concentration. The data in Fig. (5) demonstrate how *Lysinbacillus fusiformis* growth was impacted by Cupper concentrations (50 - 250 ppm). The highest protein content (127.8 mg/L) was found after 24 hrs at same dose of zinc, while The maximum optical density was recorded at 50 ppm of zinc after 24 hrs of incubation. The bacterial culture`s optical density and protein content clearly decreased at concentrations higher than this concentration. The outcomes are in a great match with Haque, et al. [79] and Wani et al. [75].

### Metal removal efficiency

As described in Table (2), the equilibrium adsorption capacity (qₑ) increased gradually as the initial metal concentration increased from 50 to 250 mg L^−1^, reaching 110.0 ± 1.2 mg g^−1^ for Cu and 108.0 ± 1.3 mg g^−1^ for Zn. Removal efficiencies remained high (80–98%), with a slight decline at higher concentrations due to partial saturation of adsorption sites. This pattern shows that the adsorbent has enough active sites to manage growing loads and has high affinity and capacity for both metals. Despite the slight decrease in removal percentage, the steady increase in qₑ indicates effective mass transfer from solution to the adsorbent surface, which is compatible with a successful adsorption process. Overall, these findings confirm the material's application in real industrial settings by demonstrating its potential for useful wastewater remediation and its efficacy across a wide range of metal concentrations. The continuous increase in qₑ without reaching a clear plateau within the studied concentration range indicates that the adsorption sites were not fully saturated, suggesting a high adsorption potential of the prepared adsorbent. These results confirm that the adsorption process is concentration-dependent and governed by the availability of active sites as well as the concentration gradient between the bulk solution and the adsorbent surface.

**Table 2:** Removal efficiency, and capacity of Cu and Zn at varying initial concentrations

Metal	Initial (mg/L)	Final (mg/L)	Removal (%)	qₑ (mg/g)
Cu	50	1.0 ± 0.2	98.0 ± 0.5	24.5 ± 0.4
Zn	50	4.0 ± 0.3	92.0 ± 0.7	23.0 ± 0.5
Cu	100	5.0 ± 0.3	95.0 ± 0.6	48.0 ± 0.6
Zn	100	10.0 ± 0.4	90.0 ± 0.7	46.0 ± 0.7
Cu	150	12.0 ± 0.5	92.0 ± 0.7	71.0 ± 0.8
Zn	150	18.0 ± 0.6	88.0 ± 0.8	69.0 ± 0.9
Cu	200	20.0 ± 0.7	90.0 ± 0.8	92.0 ± 1.0
Zn	200	30.0 ± 0.8	85.0 ± 0.9	90.0 ± 1.0
Cu	250	35.0 ± 0.9	86.0 ± 1.0	110.0 ± 1.2
Zn	250	50.0 ± 1.0	80.0 ± 1.1	108.0 ± 1.3

### Adsorption isotherm study

The adsorption equilibrium data for Cu^2^⁺ and Zn^2^⁺ onto the bacterial surface were analyzed using Freundlich and Langmuir isotherm models (Table 3). The Freundlich constants showed n values of 2.38 for Cu and 2.22 for Zn, indicating favorable adsorption (1 < n < 10) and confirming the heterogeneous nature of the bacterial cell surface. The Kf values also suggest strong affinity between metal ions and the functional groups present on the bacterial biomass.

**Table 3:** Langmuir and Freundlich adsorption isotherm constants for Cu and Zn biosorption onto bacterial surface

Metal	Kf	n	Model
Cu	24	2.38	Freundlich
Zn	21	2.22	Freundlich
Metal	**qm (mg/g)**	**KL**	**Model**
Cu	120	0.04	Langmuir
Zn	115	0.035	Langmuir

The Langmuir model gave maximum adsorption capacities (qm) of 120 mg/g for Cu and 115 mg/g for Zn, indicating high biosorption capacity and suggesting monolayer adsorption on active binding sites. The obtained KL values further confirm the strong interaction between metal ions and the bacterial surface. The good agreement of both models indicates that the biosorption process involves heterogeneous surface binding with possible monolayer formation on bacterial cell wall functional groups.

### Removal efficiency in synthetic aqueous solutions versus real wastewater

The removal efficiency of Cu and Zn was evaluated using bacterial biomass as a biosorbent in both synthetic aqueous solutions and real wastewater samples spiked with 50 mg/L of the target metals (Table 4). The obtained results demonstrate the strong capability of bacterial biomass to remove heavy metals from aqueous systems, although the efficiency varies depending on the water matrix. In the synthetic samples, the initial concentrations of Cu and Zn before spiking were negligible. After spiking with 50 mg/L and treatment with bacterial biomass, the final concentration of Cu decreased to 1.0 ± 0.2 mg/L, corresponding to a removal efficiency of 98 ± 0.5%. In the case of Zn, the final concentration reached 4.0 ± 0.3 mg/L, resulting in a removal efficiency of 92 ± 0.7%. The high removal efficiencies observed in the synthetic system indicate the strong biosorption capacity of the bacterial biomass toward heavy metal ions. Bacterial cell walls contain a variety of functional groups such as carboxyl, hydroxyl, phosphate, and amino groups, which can effectively bind metal ions through different mechanisms including ion exchange, complexation, and electrostatic attraction. The higher removal efficiency observed for Cu compared with Zn may be attributed to the stronger affinity of Cu^2^⁺ ions toward these functional groups. Copper ions generally exhibit higher binding constants with biological ligands, which enhance their interaction with the negatively charged components of the bacterial cell wall. Additionally, differences in ionic properties such as hydration energy and electronegativity may contribute to the preferential biosorption of Cu over Zn [79]. In contrast, the wastewater samples initially contained 0.04 mg/L of Cu and 0.09 mg/L of Zn, indicating slightly presence of background contamination in the wastewater matrix. After spiking till 50 mg/L and treatment using bacterial biomass, the final concentrations were 5.0 ± 0.4 mg/L for Cu and 6.0 ± 0.5 mg/L for Zn, corresponding to removal efficiencies of 88 ± 1.0% and 85 ± 1.2%, respectively. Although the bacterial biosorbent maintained relatively high removal efficiencies in the wastewater samples, the performance was slightly lower than that observed in the synthetic solutions. This decrease can be attributed to the complex composition of real wastewater, which contains a variety of dissolved ions and and organic compounds.These components may interfere with the biosorption process in several ways. Competing cations such as Ca^2^⁺, Mg^2^⁺, and Na⁺ can compete with Cu^2^⁺ and Zn^2^⁺ ions for the active binding sites on the bacterial cell surface. In addition, dissolved organic matter may form metal–organic complexes, reducing the availability of free metal ions for biosorption. Suspended particles may also partially block the active sites on the bacterial biomass, further decreasing the overall removal efficiency [80, 81]. Across both synthetic and wastewater systems, Cu removal was consistently higher than Zn removal. This trend suggests that the bacterial biomass exhibits greater selectivity toward Cu ions. Such selectivity has been reported in many biosorption studies and is mainly related to differences in metal ion affinity, coordination chemistry, and interaction with functional groups present on microbial cell surfaces [82]. The structural composition of bacterial cell walls, including peptidoglycan layers and extracellular polymeric substances (EPS), provides multiple binding sites that facilitate the uptake of heavy metals. These components can significantly enhance the biosorption capacity of bacterial biomass, making it an effective and environmentally friendly biosorbent. The results demonstrate that bacterial biomass is a highly efficient biosorbent for removing Cu and Zn from aqueous systems. Even in the more complex wastewater matrix, removal efficiencies remained above 85%, highlighting the potential of bacterial biosorption as a sustainable and cost-effective approach for heavy metal remediation.

**Table 4:** Removal efficiency in synthetic aqueous solution and real wastewater

Sample	Metal	Without spiking (mg/L)	Spiked (mg/L)	Final conc. (mg/L)	Removal (%)
Synthetic	Cu	0	50	1.0 ± 0.2	98 ± 0.5
Synthetic	Zn	0	50	4.0 ± 0.3	92 ± 0.7
Wastewater	Cu	0.04	50	5.0 ± 0.4	88 ± 1.0
Wastewater	Zn	0.09	50	6.0 ± 0.5	85 ± 1.2

### Scanning Electron Microscope (SEM)-Energy-dispersive X-Ray (EDX) and TEM

**Fig. 6:**
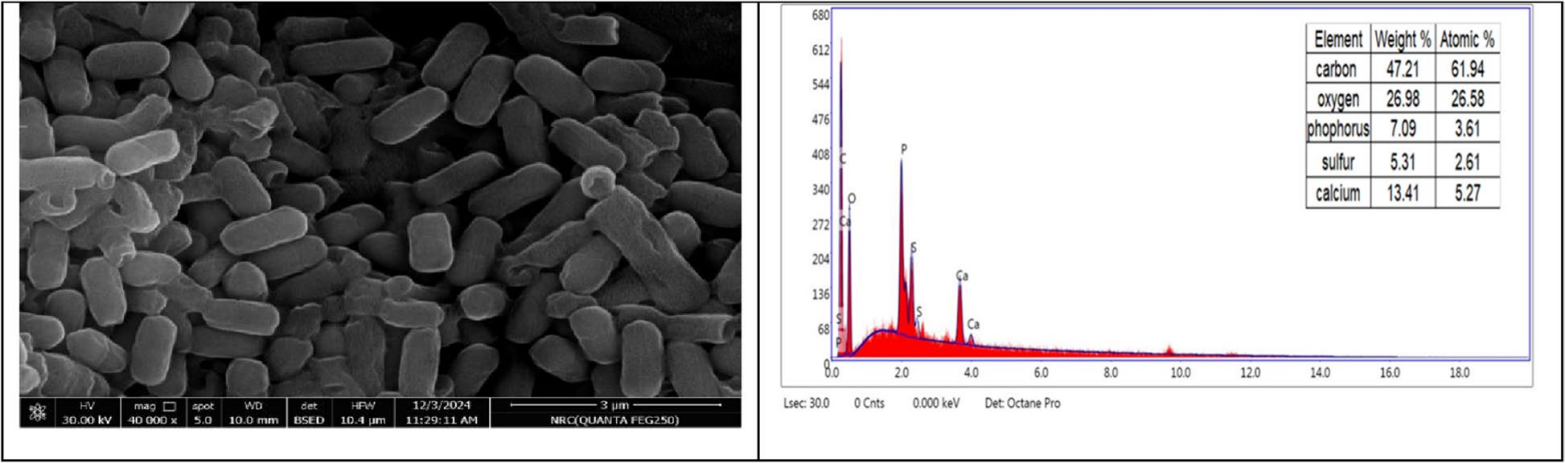
SEM–EDX micro-image exhibits the control cells of *L. fusifsormis* SKT23 (Roded health cells with smooth surfaces)

**Fig. 7:**
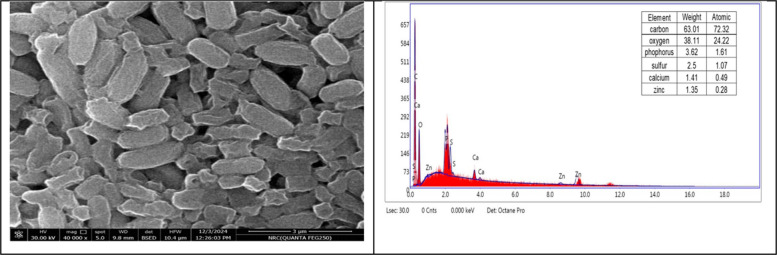
SEM–EDX micro-image exhibits the Zn biosorbent cells of *L.fusif*sormis SKT23 (the cells have shrunk and become smaller, their surface is more brilliant and not entirely rod-shaped, and there are some deposits on the borders of the bacterial cells)

**Fig. 8:**
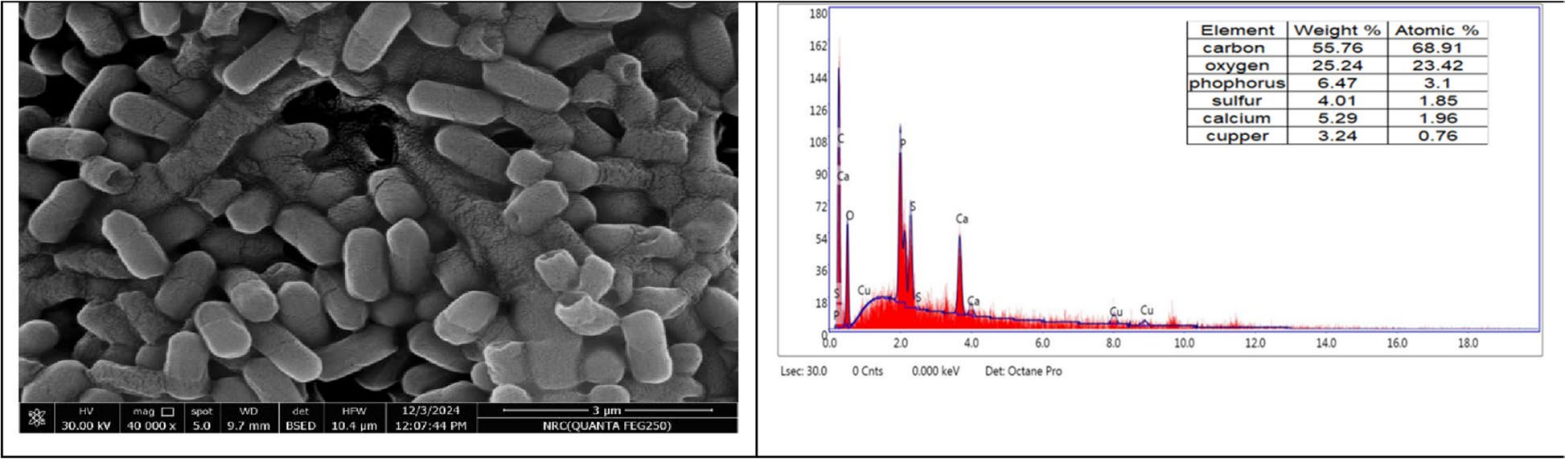
SEM–EDX micro-image exhibits the Cu biosorbent cells of *L. fusif*sormis SKT23 (some cells have slits and depressions on their surface, and some deposits and clusters were found adjacent with bacterial cells)

SEM was used in our investigation to see how the biosorption of metal ions on the bacterial cell surface would change the morphology of the cell surface, while the EDX analysis to ascertain the insertion of the studied metal ions (Cu & Zn) into the cell wall after the biosorption process. Fig. (6) demonstrates the appearance of the control cells that are rod-shaped and of the entire margins of typical elemental analysis. Fig. (7) shows the alteration in bacterial cell size that was treated with Zn; cells became aggregate and shrunken and had uneven borders with extra Zn metals on their surface. Fig. (8) shows that the Cu-treated cells developed surface wrinkles and rough as well as other abnormalities, including featuring depression on their surface and additional Cu elements.

**Fig. 9:**
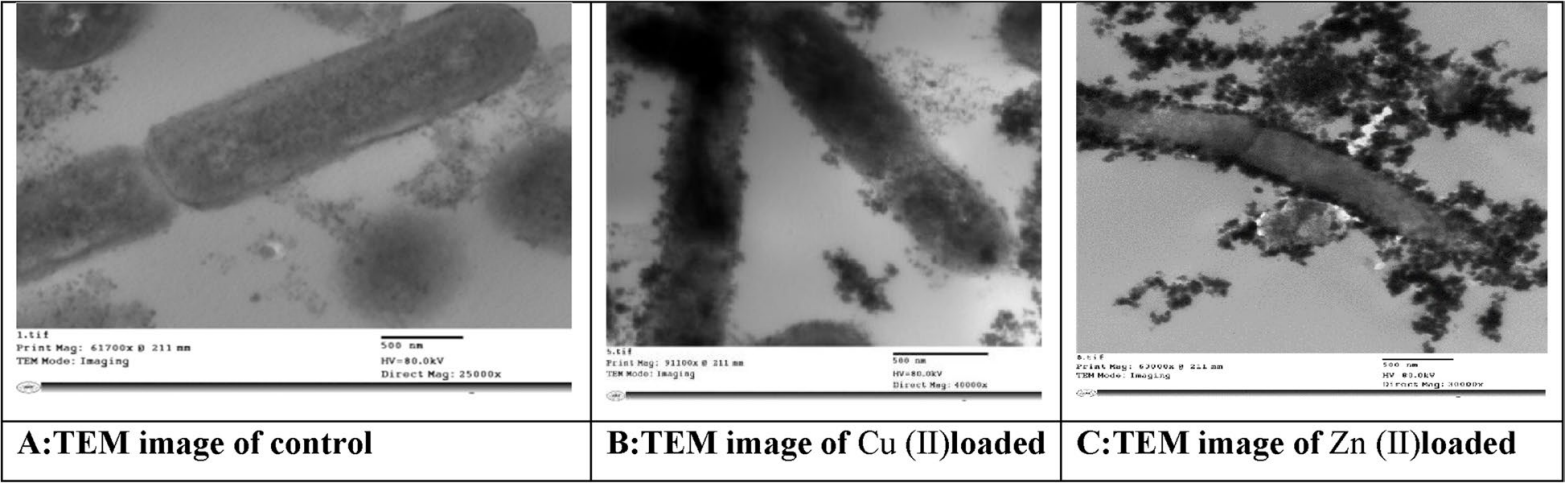
TEM images of *L. fusif*sormis SKT23

To examine the metal adhesion on the bacterial cell surface, EDX elemental analysis was offered. The insertion of the analysed metal ions (Cu & Zn) into the cell wall of *L. fusiformis* strain SKT23 following the biosorption process was verified in this investigation using EDX analysis. Biosorption took place in bacterial cells on the cell wall surface that has functional groups like hydroxyl, carboxyl, and sulphate. The metallic ions' varying affinities for the functional groups of the polysaccharides in the bacterial cell walls were the cause of the variance in Cu (II) and Zn (II) biosorption. In this study, SEM was used to analyze the morphological variations of *Lysinbacillus* with and without Zn and Cu. In the medium devoid of heavy metals, it demonstrates that bacteria grew with intact morphological features and a smooth round shape and a soft and normal rod-like appearance. On the other hand, cells exposed to heavy metals exhibit highly distorted morphological features, cell wall disruption, and aggregate formation of cells. In the presence of Cu, the surface of the bacterial cells exhibits typical corrosion characteristics, such as pits and grooves; they also become uneven and rough, fractured, and appear more brilliant. These alterations were related to structural changes in bacterial cells following Cu biosorption, which were seen to be more flattened and irregularly shaped than cells before Cu biosorption. Arivalagan et al. [65] and Khan et al. [83] have previously shown changes in bacterial cell structure in response to metal ions. In contrast to the control, the addition of Zn caused the cells to become twisted with stretched cell size and may arrange themselves together to protect from metal stress, and the broken cell surface became rough, wrinkled and permeable.These results supported the results of Krishnamurthi et al. [84] and Rajasekar et al. [85]. Cu and Zn toxicity is indicated by the noticeable morphological changes, deformation, and severe membrane damage. Furthermore, cell debris may be the release of an exopolysaccharide matrix that shields cells from metals or retains metals by adsorption, preventing their buildup within the cells [86]. EDX spectra consistently demonstrated metal deposition on the cell surface. EDX analysis demonstrates the insertion of Cu (II) and Zn (II) peaks in treated samples. Our findings are also in consistent with those of Matilda et al. [87]. The TEM micrographs of *Lysinbacillus fusif*sormis before and after Zn & Cu (II) biosorption are displayed in Fig. (9). In untreated biomass, it is evident that the cells are naked and have a clean, smooth surface. In sediments deposited inside the cytoplasm and on the cell surface of the Zn- and Cu (II)-treated *L. fusiformis*, Zn and Cu (II) ions were observed to be some dark patches of electron-dense particles that distributed irregularly, indicating the HM accumulation and precipitation. During the bioremoval of metal study, researchers such as Muñoz et al. [88] and Yan et al. [89] also noted intracellular metal buildup.

## Conclusion

*Lysinibacillus fusiformis* SKT23 demonstrates exceptional tolerance to Cu and Zn and efficiently removes these metals from both synthetic and real wastewater through robust adsorption mechanisms. Its ability to transform, precipitate, and immobilize metals, coupled with high removal efficiencies (85–98%) and strong monolayer adsorption behavior, underscores its practical potential as a sustainable and cost-effective bioremediation agent for heavy metal-contaminated water.

## Data Availability

The datasets generated and/or analyzed during the current study are available in the NCBI GenBank repository under the accession number PX488931.1.
